# High versus low-voltage pulsed radiofrequency of the suprascapular nerve for chronic shoulder pain: A randomized pilot clinical trial

**DOI:** 10.1016/j.inpm.2026.100805

**Published:** 2026-07-08

**Authors:** Joel Ribeiro Pires Costa, Francisco Javier Juan García, Daniel García Hernández, Hugo Rodríguez Rodríguez, Ana Belén Rodrigo Escudero, Manuela Barrio Alonso, Rubén Ouviña Arribas

**Affiliations:** Department of Physical Medicine and Rehabilitation, Área Sanitaria de Vigo, Vigo, Spain

**Keywords:** Chronic shoulder pain, Suprascapular nerve, Pulsed radiofrequency, High voltage, Interventional rehabilitation

## Abstract

**Background:**

Chronic shoulder pain refractory to conservative treatment remains a common and challenging clinical condition. Pulsed radiofrequency (PRF) of the suprascapular nerve (SSN) is a well-established interventional technique, typically applied at 45 V. Emerging evidence suggests that higher voltages may enhance clinical outcomes. This exploratory pilot study aimed to compare changes in pain and function after high-voltage (100 V) versus low-voltage (45 V) PRF of the SSN.

**Methods:**

A prospective, randomized, single-center, assessor-blinded pilot clinical trial was conducted. Twenty patients with chronic rotator cuff tendinopathy, baseline Visual Analog Scale (VAS) ≥7, and refractory to conservative treatment were included. Patients were randomly assigned to receive PRF of the SSN at either 45 V or 100 V, with identical technical parameters (360 s, 42 °C). Pain (VAS) and function (QuickDASH) were evaluated at baseline, 1 month, and 6 months.

**Results:**

The 100 V group showed larger observed reductions in pain compared with the 45 V group at both 1 month (between-group difference: 2.7 points; p = 0.011) and 6 months (between-group difference: 2.46 points; p = 0.022). A similar pattern was observed for QuickDASH scores. Improvements in the 45 V group were smaller and did not reach statistical significance. No serious procedure-related adverse events were recorded.

**Conclusions:**

In this exploratory pilot trial, high-voltage (100 V) PRF of the suprascapular nerve was associated with larger improvements in pain and function than 45 V PRF, although efficacy findings remain exploratory and require confirmation in larger controlled trials. No serious procedure-related adverse events were observed.

## 1 Introduction

Chronic shoulder pain is one of the most prevalent musculoskeletal conditions and represents a significant cause of long-term disability, particularly in older populations. It is frequently related to subacromial disorders, including rotator cuff tendinopathy and subacromial pain syndrome. Initial management is typically conservative and includes pharmacological treatment, therapeutic exercise, activity modification, and image-guided injections; however, a substantial proportion of patients remain symptomatic despite these interventions [1]. As a result, chronic shoulder pain continues to be a relevant source of disability, especially in elderly individuals [2].

The suprascapular nerve (SSN), arising from the upper trunk of the brachial plexus (C5–C6), plays a key role in the pathophysiology of shoulder pain, providing approximately 70% of the sensory innervation to the glenohumeral and acromioclavicular joints. After exiting the plexus, the SSN passes through the suprascapular notch beneath the transverse ligament, a region particularly susceptible to mechanical compression and traction-related injury. In addition to its sensory contribution, it provides motor innervation to the supraspinatus and infraspinatus muscles, making it a relevant target in both pain modulation and functional impairment [3].

Suprascapular nerve block has been widely used as a minimally invasive treatment for chronic shoulder pain, providing effective and sustained analgesia in selected patients. The use of ultrasound guidance allows real-time visualization of anatomical structures, improving procedural accuracy and safety while reducing the risk of complications and the need for repeated needle repositioning. Although some comparative studies have not demonstrated significant differences in pain outcomes compared with landmark-based techniques, ultrasound offers clear advantages in terms of safety and reproducibility [4].

In patients with refractory pain, radiofrequency techniques have emerged as a valuable therapeutic option. Radiofrequency can be applied in continuous (thermal) or pulsed modes, the latter being considered a neuromodulatory technique that avoids neural destruction by maintaining temperatures below 42 °C. Pulsed radiofrequency (PRF) of the SSN has been increasingly used in various shoulder conditions, including rotator cuff tendinopathy, adhesive capsulitis, glenohumeral osteoarthritis, and persistent postoperative pain, particularly when pain limits rehabilitation. Reported adverse effects include vasovagal reactions, transient neuritis, pneumothorax, hematoma, and local infection [5].

PRF outcomes are influenced by multiple technical parameters, including electrode characteristics, duration, temperature, and applied voltage [3]. Conventional protocols typically use voltages around 45 V. However, recent evidence suggests that higher voltages may enhance the electric field effect and improve analgesic outcomes without increasing adverse events. A recent observational study evaluating high-voltage (100 V) PRF of the SSN reported promising results in terms of pain and functional improvement, although the absence of a control group limits the interpretation of its findings [6]. In this context, optimizing PRF technical parameters may represent a relevant strategy to improve clinical outcomes in this clinical setting.

The aim of this exploratory pilot study was to compare changes in pain and function after high-voltage (100 V) versus low-voltage (45 V) PRF of the suprascapular nerve in patients with chronic shoulder pain secondary to rotator cuff tendinopathy. Specifically, the study evaluated pain reduction using the Visual Analog Scale (VAS), functional improvement using the QuickDASH questionnaire at 1 and 6 months, and the occurrence of procedure-related adverse events.

## 2 Materials and methods

### 2.1 Study design

A prospective, randomized, single-center, controlled clinical trial with blinding of patients and outcome assessors was conducted. Due to the lack of previous comparative studies evaluating different voltage settings for pulsed radiofrequency (PRF) of the suprascapular nerve (SSN), this study was designed as an exploratory pilot trial with a convenience sample of 20 patients. The study was not powered for efficacy, and all efficacy-related analyses were considered exploratory. Group allocation was performed using a random allocation process with an intended 1:1 ratio. Given the small sample size, a slight imbalance in final allocation occurred by chance, resulting in 12 patients in the 100 V group and 8 in the 45 V group.

### 2.2 Study population and eligibility criteria

The study population consisted of patients treated at the Shoulder Unit of the Department of Physical Medicine and Rehabilitation at Hospital Meixoeiro (Vigo, Spain) between January and March 2025. A total of 20 patients with chronic rotator cuff tendinopathy of more than 3 months’ duration and baseline pain intensity ≥7 on the Visual Analog Scale (VAS) were included. The diagnosis of chronic rotator cuff tendinopathy was established based on compatible clinical history, physical examination, and ultrasound findings.

Inclusion criteria were: age ≥18 years, both sexes, acceptance of the interventional treatment, ability to provide informed consent, and a positive response to diagnostic and confirmatory suprascapular nerve block defined as a ≥50% reduction in pain, confirming a neural origin of pain.

Exclusion criteria included: pain secondary to other etiologies, local or systemic infection, uncorrected coagulopathy, known allergy to co-administered drugs, previous interventional procedures within the last 6 months, decompensated chronic diseases, presence of implanted electrical devices, refusal to participate, or withdrawal of informed consent.

### 2.3 Interventional procedure

**Fig. 1:**
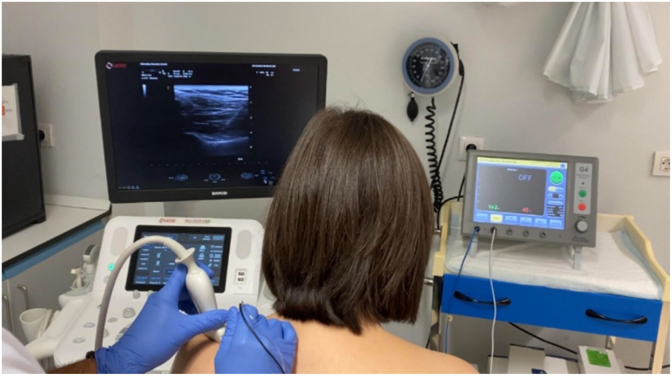
Ultrasound-guided pulsed radiofrequency of the suprascapular nerve. Authors' own images.

**Fig. 2:**
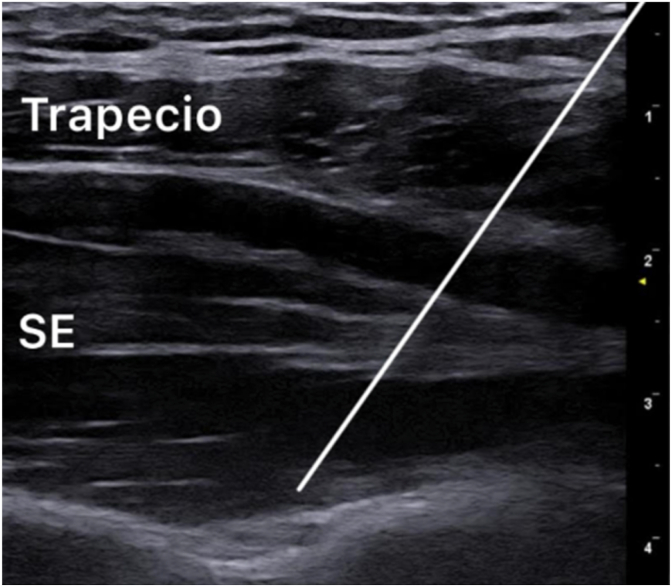
Ultrasound image of the suprascapular notch showing the trapezius and supraspinatus (SS) muscles, and needle trajectory toward the suprascapular nerve. Authors' own images.

All procedures were performed under ultrasound guidance using an Esaote MyLab™ X8 eXP system with a high-frequency linear probe (4–15 MHz). Patients were positioned in a seated position with the arms at rest. The transducer was placed over the supraspinous fossa, parallel to the scapular spine, identifying the trapezius and supraspinatus muscles, the suprascapular artery, and the transverse scapular ligament to locate the suprascapular notch (Fig. 1, Fig. 2).

Sensory stimulation was used to reproduce concordant paresthesia within the target territory without atypical radiation, while motor stimulation elicited contraction of the supraspinatus and infraspinatus muscles, confirming proximity to the nerve while avoiding non-target motor fibers. Sensory stimulation was performed at ≤0.5 V, and motor stimulation was accepted at up to twice the sensory threshold. After anatomical identification using color Doppler, confirmation of appropriate sensory and motor responses, and negative aspiration, 4 mL of 2% mepivacaine was administered. PRF was then applied using standard parameters (42 °C, 360 s), consistent with commonly described protocols for peripheral nerves.

**Fig. 3:**
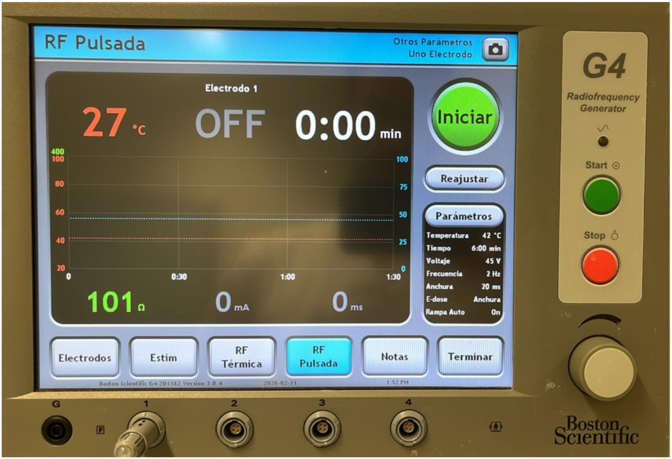
Radiofrequency generator display during PRF application at 45 V (42 °C, 360 s). Authors' own images.

**Fig. 4:**
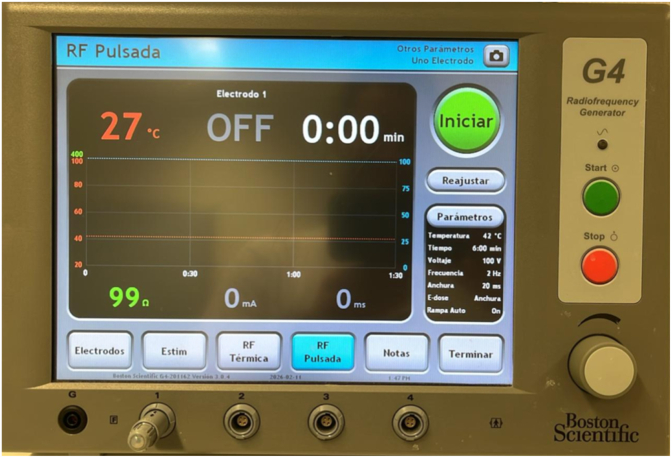
Radiofrequency generator display during PRF application at 100 V (42 °C, 360 s). Authors' own images.

Patients were randomly assigned to Group A (45 V) or Group B (100 V) (Fig. 3, Fig. 4). Procedures were performed using a G4™ RF Generator (Cosman®, Marlborough, MA, USA) with 22-gauge radiofrequency cannulas (6 cm length, 5 mm active tip) with temperature control.

### 2.4 Outcome measures and follow-up

Demographic variables, including age, sex, and affected side, were recorded. The primary outcome measures were pain intensity assessed by the Visual Analog Scale (VAS, 0–10) and functional status assessed using the QuickDASH questionnaire. The QuickDASH is a validated 11-item instrument measuring upper limb disability, scored from 0 to 100, with higher scores indicating greater disability [7]. Both outcomes were assessed at three time points: baseline (pre-intervention), 1 month, and 6 months post-procedure. Follow-up assessments were performed in person by physicians blinded to treatment allocation, who were not involved in the interventional procedures.

### 2.5 Statistical analysis

All randomized patients were included in the analysis, as no losses to follow-up occurred. Normality of quantitative variables was assessed using the Shapiro–Wilk test. Continuous variables were expressed as mean and standard deviation (SD). Baseline between-group comparisons for demographic variables were performed using independent-samples Student's t-test for continuous variables and Fisher's exact test for categorical variables. Within-group comparisons (baseline vs follow-up) were performed using paired Student's t-tests. Between-group differences (45 V vs 100 V) were analyzed using change scores (Δ), defined as follow-up value minus baseline (Δ at 1 month and Δ at 6 months), and compared using independent-samples Student's t-tests. Homogeneity of variances was assessed using Levene's test. Results were reported as mean differences with 95% confidence intervals (95% CI). A p-value <0.05 was considered statistically significant. Statistical analyses were performed using IBM SPSS Statistics version 27.0. Responder analysis was also performed. Treatment success was defined as ≥50% pain relief from baseline VAS, a commonly used threshold in pain medicine [8]. The proportion of patients achieving treatment success was calculated for each group at 1 and 6 months, together with corresponding 95% confidence intervals (95% CI). Confidence interval overlap between groups was assessed descriptively.

### 2.6 Ethical considerations

The study was approved by the Research Ethics Committee of Galicia (CEIm-Galicia; approval number 2025/313) and conducted in accordance with the principles of the Declaration of Helsinki.

All participants provided written informed consent prior to inclusion. Data confidentiality and pseudonymization were ensured in accordance with Regulation (EU) 2016/679 and Spanish Organic Law 3/2018. Participation in the study did not affect access to standard clinical care.

## 3 Results

A total of 20 patients were assessed for eligibility, all of whom met the inclusion criteria and were randomized. All patients completed the 6-month follow-up and were included in the final analysis. The study population comprised 20 patients, of whom 75% were female (n = 15) and 25% male (n = 5), with a mean age of 67.8 years. Twelve patients (60%) were allocated to the 100 V PRF group and eight (40%) to the 45 V group. In the 100 V group, 75% were female (n = 9) with a mean age of 72.9 years, whereas in the 45 V group, 75% were female (n = 6) with a mean age of 60.1 years. Baseline characteristics of both groups are summarized in Table 1.

**Table 1:** Demographic characteristics of the overall sample and by treatment group (100 V vs 45 V).

	Total (n = 20)	100 V (n = 12)	45 V (n = 8)	p-value
**Sex**	15 female (75%)	9 female (75%)	6 female (75%)	1.000
	5 male (25%)	3 male (25%)	2 male (25%)	
**Age (years)**	67.8 ± 13.6	72.9 ± 9.5	60.1 ± 15.7	0.035

Regarding pain outcomes, the 100 V group showed a significant reduction in VAS scores from baseline (mean 7.92) to 1 month (mean 3.58), corresponding to a decrease of 4.33 points, and a reduction of 3.58 points at 6 months (mean 4.33), both statistically significant (p < 0.001). In contrast, the 45 V group (baseline mean 7.88) did not demonstrate statistically significant reductions at 1 month (mean 6.25) or at 6 months (mean 6.75) (p > 0.05) (Table 2). Exploratory between-group analysis showed that the observed reduction in VAS at 1 month was larger in the 100 V group (mean reduction 4.33 points) compared with the 45 V group (mean reduction 1.63 points), resulting in a between-group difference of 2.71 points (95% CI 0.71 to 4.71; p = 0.011). Similarly, at 6 months, the observed reduction was larger in the 100 V group (3.58 points) compared with the 45 V group (1.13 points), with a between-group difference of 2.46 points (95% CI 0.39 to 4.53; p = 0.022) (Table 3). Responder analysis using ≥50% pain relief from baseline VAS as the criterion for treatment success showed higher responder rates in the 100 V group than in the 45 V group at both 1 month (66.7% vs 25.0%) and 6 months (50.0% vs 12.5%) (Table 6).

**Table 2:** VAS scores (mean ± SD) at baseline, 1 month, and 6 months by treatment group, and within-group changes from baseline at 1 and 6 months. Paired Student's t-test.

Group	VAS baseline (mean ± SD)	VAS 1 month (mean ± SD)	VAS 6 months (mean ± SD)	Δ 1 month (95% CI)	Δ 6 months (95% CI)	Within-group p-value
**100 V (n=12)**	7.92 ± 1.24	3.58 ± 2.15	4.33 ± 2.31	−4.33 (−5.67 to −3.00)	−3.58 (−4.98 to −2.19)	<0.001
**45 V (n=8)**	7.88 ± 1.13	6.25 ± 2.12	6.75 ± 2.38	−1.63 (−3.35 to 0.10)	−1.13 (−2.88 to 0.63)	>0.05

**Table 3:** Between-group comparison (100 V vs 45 V) of mean change in VAS at 1 and 6 months from baseline. Independent-samples Student's t-test (Levene's test for equality of variances).

VAS	100 V	45 V	Δ 100V-45V (95% CI)	p-value
**Δ 1 month**	−4.33	−1.63	2.71 (0.71 to 4.71)	0.011
**Δ 6 months**	−3.58	−1.13	2.46 (0.39 to 4.53)	0.022

Functional outcomes assessed by QuickDASH showed a similar pattern. In the 100 V group, QuickDASH scores improved from a baseline mean of 70.85% to 35.73% at 1 month (mean reduction 35.12%) and to 40.49% at 6 months (mean reduction 30.36%), both statistically significant (p < 0.001). In contrast, the 45 V group (baseline mean 65.61%) did not show statistically significant improvements at 1 month (60.15%) or at 6 months (59.16%) (p > 0.05) (Table 4). Exploratory between-group comparisons revealed that the observed reduction in QuickDASH at 1 month was larger in the 100 V group (35.12%) compared with the 45 V group (5.46%), resulting in a between-group difference of 29.66% (95% CI 18.44 to 40.87; p < 0.001). At 6 months, the reduction remained larger in the 100 V group (30.35%) compared with the 45 V group (6.45%), with a between-group difference of 23.90% (95% CI 8.33 to 39.48; p = 0.005) (Table 5).

**Table 4:** QuickDASH scores (mean ± SD) at baseline, 1 month, and 6 months by treatment group, and within-group changes from baseline at 1 and 6 months. Paired Student's t-test.

Group	QuickDASH baseline (mean ± SD)	QuickDASH 1 month (mean ± SD)	QuickDASH 6 months (mean ± SD)	Δ 1 month (95% CI)	Δ 6 months (95% CI)	Within-group p-value
**100 V (n=12)**	70.9% ± 9.1%	35.7% ± 12.8%	40.5% ± 19.0%	−35.1% (−42.2 to −28.1)	−30.4% (−40.7 to −20.0)	<0.001
**45 V (n=8)**	65.6% ± 11.8%	60.1% ± 15.3%	59.2% ± 17.5%	−5.5% (−16.01 to 5.08)	−6.5% (−19.92 to 7.02)	>0.05

**Table 5:** Between-group comparison (100 V vs 45 V) of mean change in QuickDASH at 1 and 6 months from baseline. Independent-samples Student's t-test (Levene's test for equality of variances).

QuickDASH	100 V	45 V	Δ 100V-45V (95% CI)	p-value
**Δ 1 month**	−35.1%	−5.5%	29.66% (18.44 to 40.87)	<0.001
**Δ 6 months**	−30.4%	−6.5%	23.90% (8.33 to 39.48)	0.005

**Table 6:** Proportion of patients achieving treatment success (≥50% pain relief from baseline VAS). 95% confidence intervals were calculated using the exact Clopper–Pearson method.

Follow-up	100 V	95% CI	45V	95% CI
**1 month**	8/12 (66.7%)	34.9 to 90.1	2/8 (25.0%)	3.2 to 65.1
**6 months**	6/12 (50.0%)	21.1 to 78-9	1/8 (12.5%)	0.3 to 52.7

## 4 Discussion

The present exploratory pilot study provides preliminary evidence regarding the potential influence of voltage settings in pulsed radiofrequency (PRF) of the suprascapular nerve for patients with chronic shoulder pain secondary to rotator cuff tendinopathy refractory to conservative treatment. The main findings suggest that high-voltage PRF (100 V) was associated with larger observed improvements in pain, as measured by the Visual Analog Scale (VAS), and in functional status, as assessed by the QuickDASH questionnaire, with benefits maintained at both 1 and 6 months after the procedure. These findings suggest that optimization of PRF technical parameters may represent a relevant strategy to improve outcomes in this patient population. From a clinical perspective, the magnitude of effect observed in the 100 V group exceeded the minimal clinically important difference (MCID) reported for both scales, generally considered to be approximately 2 points for VAS and a clinically meaningful change for QuickDASH. In addition, responder analysis also favored the 100 V protocol, with higher rates of treatment success (≥50% pain relief) at both follow-up assessments. Although confidence intervals overlapped, likely reflecting the limited sample size of this pilot study, the observed responder rates consistently favored the high-voltage protocol.

Recently, García-Amigo et al. [6] published a prospective study evaluating high-voltage (100 V) PRF of the suprascapular nerve in patients with chronic shoulder pain, reporting significant reductions in pain and functional disability at 6 months. Their findings provide relevant support for the effectiveness of high-voltage PRF in this setting. However, the absence of a comparator group limits the ability to determine whether the observed improvement was attributable specifically to the use of high voltage or to the effect of PRF itself. In this regard, our study expands upon these findings by incorporating a randomized design with a control group, allowing direct comparison between two voltage protocols under otherwise homogeneous technical conditions. Our results are consistent with these findings and suggest that, in this small exploratory sample, 100 V PRF may be associated with larger improvements than the 45 V protocol. However, these observations require confirmation in adequately powered randomized trials.

From a mechanistic perspective, the greater effectiveness observed with high-voltage PRF may be explained by the generation of a stronger electric field around the active tip of the electrode, thereby enhancing neuromodulatory effects on nociceptive fibers without causing thermal injury when sublesive temperatures are maintained (<42 °C). Previous studies have shown that increasing voltage may enlarge the electric field and intensify its biological effects, promoting modulation of nociceptive transmission and neuronal plasticity, which may translate into a more robust and longer-lasting analgesic response [[9], [10], [11], [12], [13]].

By contrast, the 45 V group showed a trend toward improvement in both pain and function over follow-up, although the magnitude of change was smaller and did not reach statistical significance. This finding is consistent with the existing literature on PRF of the suprascapular nerve, which has reported variable clinical benefit and, in some cases, more modest or shorter-lasting effects [[14], [15], [16], [17], [18], [19]]. Our results suggest that low-voltage PRF may induce partial neuromodulation in some patients, although improvements in this exploratory sample appeared smaller than those observed with higher-voltage protocols.

With regard to safety, no serious procedure-related adverse events were observed in either group. Although no conclusions regarding safety can be drawn from this small exploratory sample, these observations are consistent with previous studies and systematic reviews reporting a low incidence of complications associated with PRF of the suprascapular nerve, including high-voltage protocols [[20], [21], [22], [23], [24]]. Furthermore, García-Amigo et al. [6] also reported no relevant safety concerns with 100 V PRF.

This study has several limitations that should be acknowledged. First, the small sample size, inherent to its pilot design, limits generalizability and statistical power for subgroup analyses. Second, a statistically significant difference in mean age between groups was observed at baseline and may have acted as a potential confounding factor. Given the small exploratory sample size, adjusted analyses to account for baseline imbalances were not considered sufficiently robust and should be explored in larger adequately powered studies. Third, although the 6-month follow-up allows assessment of medium-term persistence of effect, no conclusions can be drawn regarding long-term durability. Therefore, the observed differences between groups should be interpreted as hypothesis-generating and require confirmation in larger, adequately powered controlled studies. Additionally, because only patients with chronic rotator cuff tendinopathy were included, the findings may not be generalizable to other causes of chronic shoulder pain.

## 5 Conclusions

In this exploratory pilot trial, high-voltage (100 V) pulsed radiofrequency of the suprascapular nerve was associated with larger observed improvements in pain and function compared with the standard 45 V protocol. These findings support further investigation of PRF voltage optimization in larger controlled studies. No serious procedure-related adverse events were observed.
